# Treatment of Clinical Solid Waste Using a Steam Autoclave as a Possible Alternative Technology to Incineration 

**DOI:** 10.3390/ijerph9030855

**Published:** 2012-03-09

**Authors:** Md. Sohrab Hossain, Venugopal Balakrishnan, Nik Norulaini Nik Ab Rahman, Md. Zaidul Islam Sarker, Mohd Omar Ab Kadir

**Affiliations:** 1 Department of Environmental Technology, School of Industrial Technology, Universiti Sains Malaysia, 11800 Penang, Malaysia; Email: msh.id09@student.usm.my; 2 Institute for Research in Molecular Medicine, Universiti Sains Malaysia, 11800 Penang, Malaysia; Email: venugopal@usm.my; 3 School of Distance Education, Universiti Sains Malaysia, 11800 Penang, Malaysia; Email: norulain@usm.my; 4 Department of Pharmaceutical Technology, Faculty of Pharmacy, International Islamic University Malaysia, Kuantan Campus, Bandar Indera Mahkota, 25200 Kuantan, Pahang, Malaysia; Email: zaidul@iium.edu.my

**Keywords:** autoclave, clinical solid waste, clinical solid waste management, inactivation of bacteria, treatment technology

## Abstract

A steam autoclave was used to sterilize bacteria in clinical solid waste in order to determine an alternative to incineration technology in clinical solid waste management. The influence of contact time (0, 5, 15, 30 and 60 min) and temperature (111 °C, 121 °C and 131 °C) at automated saturated steam pressure was investigated. Results showed that with increasing contact time and temperature, the number of surviving bacteria decreased. The optimum experimental conditions as measured by degree of inactivation of bacteria were 121 °C for 15 minutes (min) for Gram negative bacteria, 121 °C and 131 °C for 60 and 30 min for Gram positive bacteria, respectively. The re-growth of bacteria in sterilized waste was also evaluated in the present study. It was found that bacterial re-growth started two days after the inactivation. The present study recommends that the steam autoclave cannot be considered as an alternative technology to incineration in clinical solid waste management.

## 1. Introduction

There is growing interest in identifying a reliable technology for safe handling and disposal of clinical solid waste (CSW). CSW is any solid material generated during the diagnosis, treatment, or immunization of human beings or animals and testing of biological fluids [1]. Examples of CSW include, but are not limited to discarded surgical gloves and instruments, needles, lancets, culture, stocks and swabs, soiled or blood-soaked bandages, culture dishes and other glassware [1,2,3,4]. The management and safe disposal of CSW is problematic due to the infectious nature of the waste and its high disposal costs. Developing nations have undertaken to determine possible ways to minimize the health hazards and environmental pollution from the risk exposure of CSW disposal practices. Although, significant improvements have been achieved, further investigation is still required to define cost effective and safe disposal practices [5]. 

The risk posed by CSW to human health and the environment, which needs to be assessed, is due to the presence of pathogenic microorganisms. CSW is prescribed by many as infectious or hazardous due to the fact it can contain a great variety of pathogenic microorganisms which commonly include bacteria, viruses, fungi, and prions [5,6,7,8,9]. Personnel involved in the treatment of clinical waste are exposed to infectious agents through several routes, including skin penetration, skin contact, or the aerogenic route [5,7,8]. The most typical pathogenic bacteria in CSW are the genus *Bacillus, Staphylococci* and *Streptococci*, along with varying numbers of other common nosocomial pathogenic bacteria such as *Klebsiella*, *Salmonella*, *Proteus* and *Enterobacter* species [5,10]. However, the most prevalent bacteria found in CSW are *Staphylococcus aureus*, *Escherichia coli*, *Pseudomonas aeruginosa*, *Bacillus cereus* and *Candida albicans* [5,10,11]. Park *et al.* [11] detected a number of microorganisms, including *Pseudomonas* spp., *Lactobacillus* spp., *Staphylococcus* spp., *Micrococcus* spp., *Kocuria* spp., *Brevibacillus* spp., *Microbacterium oxydans*, and *Propionibacterium acnes* in various types of clinical wastes. Alagoz and Kocasoy [5] conducted microbiological analysis in CSW to determine the quantity of pathogenic becteria by colony count methods. Coliform bacteria, *Escherichia coli*, *Enterobacter*, *Pseudomonas* spp., *Staphylococcus aureus*, *Bacillus cereus*, *Salmonella* spp., *Legionella* and yeast and moulds were detected in clinical waste. 

Worldwide the most commonly used technology to treat CSW is incineration. The distinct advantage of incineration technology in CSW management is that it greatly reduces the volume of the waste while rendering the waste unrecognizable [12,13,14]. Conversely, incineration releases a wide variety of pollutants, including dioxins and furans, heavy metals (such as lead, mercury, and cadmium), acid gases (hydrogen chloride and sulfur dioxide), carbon monoxide, and nitrogen oxide [8,12,14]. These emissions can have serious effects on worker safety, public health and the environment [2,15,16,17,18]. Dioxin, for example, is linked to cancer, immune system disorders, diabetes, birth defects and other health related diseases [16,18]. It has been reported that clinical waste incinerators are a leading source of dioxin and mercury in the environment [16,17]. Further, the resulting waste such as fly ash and bottom ash might contain dioxins, toxic chemical and heavy metals and therefore has to be disposed as a hazardous waste [6,10,17]. In addition, there is the possibility that heat resistant pathogenic microorganisms may remain viable in stack gas and bottom ash [9,19]. Treatment cost is another key factor in the consideration of CSW disposal technology. Incineration requires high financial start-up costs and occupational capital to implement incineration facilities [2,6,10,20]. Incineration can therefore be considered as an inappropriate technology for treating CSW [6,20,21]. This has led to considerable interest in eliminating incineration due to the human health and environmental pollution concerns by identifying an acceptable alternative to incineration technology for the safe disposal of CSW. In recent years, environmentalists and policy agencies have sought to define suitable sterilization technologies to sterilize the CSW at its source prior to continuing the recycle and reuse of CSW materials [14,21,22]. Steam autoclaving has been considered under the broad umbrella of clinical waste treatment technologies during State and Territorial Association on Alternate Treatment Technologies (STAATT) III conference summary meeting [23]. Therefore, CSW treatment with a steam autoclave is receiving considerable attention as a possible alternative to incineration as well as sustainable development of CSW management with continued recycle and reuse of CSW materials from sterilized waste. 

Autoclave technology has also been widely used in healthcare facilities to decontaminate highly infectious lab waste because it is viewed as the most reliable and easily controllable process. Typically, autoclaves are used in hospitals for the sterilization of reusable medical equipment and have been proven to be very effective for that purpose. Later, the same process parameters have been applied to sterilize clinical waste with the hypothesis that an autoclave could be effective to sterilize the clinical waste as well [24]. However, evidence to support this claim is scarce. Further, the factors affecting sterilization efficiency of microorganisms in clinical waste have not been thoroughly studied. Although a few studies have documented that autoclaves effectively inactivate pathogenic microorganisms in the waste, the possible re-growth of pathogenic bacteria has been neglected. Therefore, the present study was conducted to determine the possibility of using steam sterilization as an alternative of incineration technology. The experiments were conducted on a lab scale using six common pathogenic bacteria which included three Gram positive bacteria (*i.e.*, *Bacillus subtilis*, *Streptococcus pyogenes*, *Staphylococcus aureus*) and three Gram negative bacteria (*i.e.*, *Escherichia coli*, *Psudomonas aeroginosa*, *Acinetobacter baumannii*) as test microorganisms to determine the sterility of the waste. The bacteria were chosen based on the prevalence of commonly detected bacteria during patient care in the studied hospital. The influence of the process parameters such as contact time and temperature were determined. Further, the re-growth factor of bacteria from the sterilized waste was also measured.

## 2. Experimental Section

### 2.1. Preparation of Bacteria

The bacteria used in this study were collected from the isolation section of the Department of Microbiology and Parasitology, Hospital Universiiy Sains Malaysia, Kelantan, Malaysia. The list of bacteria used in the present study is shown in Table 1. The bacteria collected were stored in a refrigerator at 4 °C temperature. The microorganisms were re-grown in selective media to obtain fresh cultures (*i.e.*, blood agar media for Gram positive bacteria and MacConkey agar media for Gram negative bacteria). From the culture growth, a single isolated colony was transferred to nutrient broth (NB) agar and incubated at 37 °C for 48 hours.

**Table 1:** Log reduction of bacteria in sterilized waste. Sterilization conditions: temperature 121 °C, time 60 minutes and pressure 15 psi.

Bacteria	Initial concentration, N_0_	Log reduction Log N_0_/N
*Escherichia coli*	2.90 × 10^8^	8.5
*Psudomonas aeroginosa*	2.92 × 10^8^	8.5
*Acinetobacter baumannii*	2.95 × 10^8^	8.5
*Staphylococcus aurous*	2.55 × 10^8^	8.4
*Streptococcus pyogenes*	1.25 × 10^8^	8.0
*Bacillus subtilis* (Vegetative form)	3.4 × 10^8^	7.5

### 2.2. Sample Preparation

The CSW materials used in this study was collected from Lam Wah Ee Hospital, one of the specialist healthcare facilities in Penang Island, Malaysia. The collected samples were sterilized in an autoclave to ensure their safe handling. Later, the sterilized waste was dried at room temperature to minimize the moisture content. Heat resistant waste materials (*i.e.*, hard plastic materials, broken glass, textile, metals, *etc*.) were then separated from the sterilized waste by manual separation. Later the waste materials were sorted in desired sizes by manual cutting between 0.5–1.0 inches. One kg of waste was taken in biohazard autoclave bag (size 12 × 24 cm; Fisher brand) for further study. The gravimetric composition of clinical solid wastes used in this study is presented in Table 2.

**Table 2:** Gravimetric composition of clinical solid wastes used in this study.

Material	% in mass (dry basis)
Hard plastic	40
Broken glass	25
Fabric	15
metal	20
Rubber	10
Total = 100	

One hundred mL of bacterial solution was prepared using 10 mL of bacterial growth in NB agar, 40 mL of sterilized glycerol and 50 mL sterilized 0.9% saline solution. The glycerol was used as a surfactant for the homogenous distribution of bacteria in the sample. The mixture of the bacteria was then added to 1 kg of waste dropwise and mixed vigorously using a glass rod. The biohazard autoclave bag was then loosely constricted by a twist-tie, a few holes made by punching the top side of the bag for the steam heat penetration into the waste, and replaced in the autoclave system for the sterilization study.

### 2.3. Inactivation of Bacteria in CSW

The inactivation of bacteria in CSW was performed using a laboratory autoclave (Model: ES-215; Tomy Seiko Co., Ltd). The influence of contact time (0, 5, 15, 30 and 60 min) and temperature (111 °C, 121 °C and 131 °C) under automated saturated steam pressure on inactivation of bacteria were determined. The pressures were found to be 8 psi, 15 psi and 27 psi for the temperatures of 111 °C, 121 °C and 131 °C, respectively. The number of surviving colonies in the sample was determined before treatment (at the time 0 min) and after the treatment using a pour-plate method. For the colony counting of bacteria, the bacterium-contaminated sample was diluted in sterilized distilled waste with a ratio of 1:5 g/mL. Next, 1 mL of contaminated diluted sample was taken for the eight fold serial dilution, from which 0.1 mL of removed for seeding on Petri dishes containing nutrient agar media using a sterile Drigalski spatula. The dishes with the culture medium were labeled and incubated at 37 °C for 48 hours prior to counting. This procedure was carried out in duplicate to determine the average bacterial colony concentration in waste. Results were expressed as the logarithm of surviving colony forming units per gram of waste (Log CFU/g). It should be noted that any bacterial colony that did not grow on the surface of the agar plate from the treated sample, would lead to overestimation of the degree of inactivation [25]. However, if there is no bacterial colony recovery from the treated sample, the total bacterial colony was considered as one colony due to the logarithmic study. All experiments were conducted twice and the average result was considered for the analysis.

The amount of bacterial colonies was calculated by the number of individual colonies that formed on the surface of agar plates. The number of colony forming units per gram of waste was calculated using the following equation [26]:


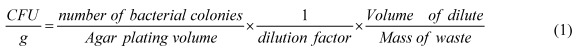


where, agar plating volume was 0.1 mL, volume of dilute was 5 mL sterilized distilled water and the mass of waste was 1 g. The log reduction of bacterial colony in per gram of sample was calculated as [27]: 


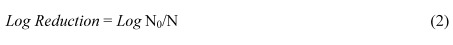


where N_0_ is the number of surviving colonies in the untreated samples, and N is the number of surviving colonies in the sterilized samples. 

### 2.4. Bacterial Re-Growth

A mixture of bacterial solution containing 10 mL of growth in NB agar of each of the studied bacteria, 40 mL of sterilized glycerol and 100 mL sterilized 0.9% saline solution, was added dropwise to 2 kg of sterilized sample and mixed vigorously using a glass rod. The biohazard autoclave bag was then loosely constricted by a twist-tie, a few holes made by punching the top side of the bag for the steam heat to penetrate into the waste. The sample was then sterilized using the optimized experimental condition determined in the preliminary studies (121 °C for 60 min). 

After sterilization, the sample was replaced in the biohazard bin with 1 L added sterilized distilled water and mixed vigorously using a glass rod. Later, the sample was stored at room temperature (25 ± 1 °C) for determining the re-growth of bacteria. The bacterial re-growth in sterilized samples was observed at 24 hour intervals for 0–7 days. For the observation of re-growth, the sample in the waste bin was shaken and 1 mL of contaminated sample taken to culture on agar media. Blood and MacKonkey agar medium was used to culture the bacteria for Gram positive and Gram negative bacteria, respectively. Gram stain reaction and other biochemical tests included catalase test, oxidase test, triple sugar iron test, used for the morphological analysis of bacteria, were conducted according to the guidelines of the manual of microbiological analysis provided by Department of Microbiology and Parasitology, Hospital Universiti Sains Malaysia. Finally, the species of bacteria was confirmed using selective Analytab Products Inc. (API) kit analysis.

## 3. Results and Discussion

**Figure 1:**
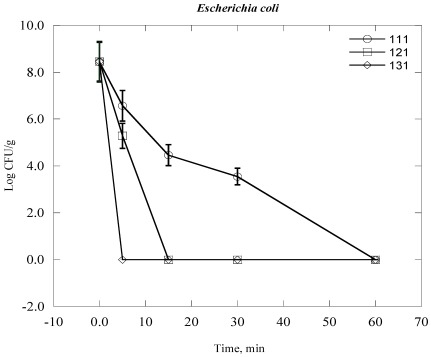
Inactivation of *E. coli* in clinical solid waste using steam sterilization. Experimental conditions: (○), 111 °C (8 psi); (□), 121 °C (15 psi) and (◊), 131 °C (27 psi).

**Figure 2:**
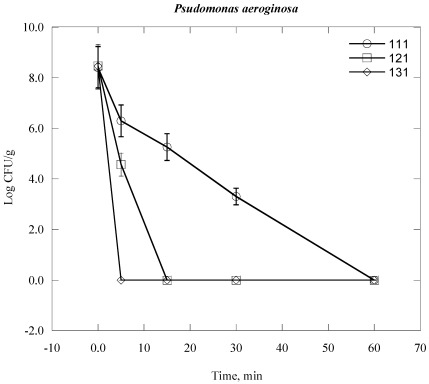
Inactivation of *P. aeruginosa* in clinical solid waste using steam sterilization. Experimental conditions: (○), 111 °C (8 psi); (□), 121 °C (15 psi) and (◊), 131 °C (27 psi).

**Figure 3:**
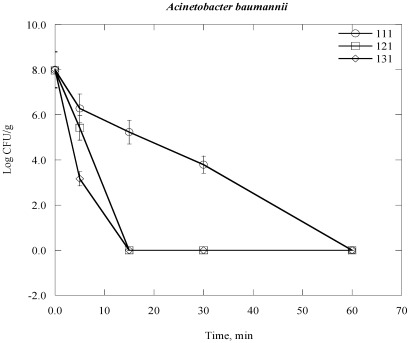
Inactivation of *Actinetobacter baumannii* in clinical solid waste using steam sterilization. Experimental conditions: (○), 111 °C (8 psi); (□), 121 °C (15 psi) and (◊), 131 °C (27 psi).

**Figure 4:**
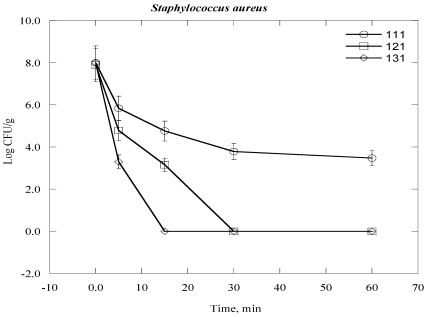
Inactivation of *Staphylococcus aureus* in clinical solid waste using steam sterilization. Experimental conditions: (○), 111 °C (8 psi); (□), 121 °C (15 psi) and (◊), 131 °C (27 psi).

**Figure 5:**
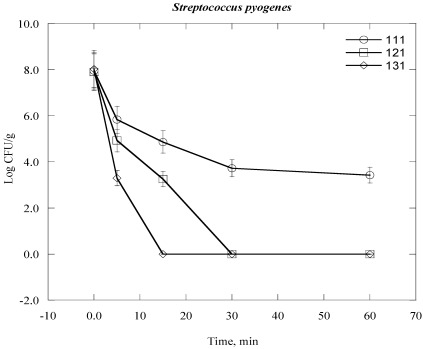
Inactivation of *Streptococcus pyogenes* in clinical solid waste using steam sterilization. Experimental conditions: (○), 111 °C (8 psi); (□), 121 °C (15 psi) and (◊), 131 °C (27 psi).

**Figure 6:**
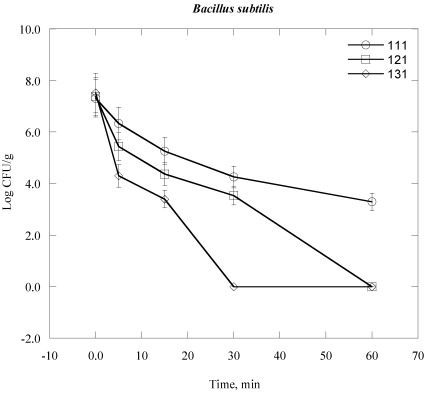
Inactivation of *Bacillus subtilis* in clinical solid waste using steam sterilization. Experimental conditions: (○), 111 °C (8 psi); (□), 121 °C (15 psi) and (◊), 131 °C (27 psi).

Steam sterilization inactives microorganisms through the application of saturated steam under pressure. It generally denotes heating in an autoclave employing saturated steam under a certain pressure to achieve the desired chamber temperature [24,28]. The process thermally damages the bacterial cell structure, including the outer and cytoplasmic membrane, and rendering the cell no longer viable. The inactivation of bacterial cell vital mechanisms depends on the bacterial cell structure, the temperature and duration of the heat exposure to which they are exposed [28]. In practical terms which means that it would take a longer time at lower temperatures to sterilize a population than at a higher temperature. For example, bacterial colony survival decreases with increasing duration of time of autoclaving at elevated temperature and automated pressure (Figure 1, Figure 2, Figure 3, Figure 4, Figure 5, Figure 6). However, noticeable differences on decrease of the Log CFU/g sample with increasing temperature were observed between Gram negative and Gram positive bacteria. Differences were also found among the Gram positive and Gram negative bacteria as well. In the case of *Escherichia coli* (*E. coli*) and *Psudomonas aeroginosa* (*P. aeroginosa*), Log CFU/g was found to decrease with increasing duration of exposure and reached almost zero at 60, 15 and 5 min for 111 °C, 121 °C and 131 °C, respectively (Figure 1 and Figure 2). The degree of inactivation at elevated temperature and duration time was different in the case of *Acinetobacter baumannii* (*A. baumannii*) compared to *E. coli* and *P. aeruginosa* (Figure 3). As we can see in Figure 3, the Log CFU/g reached in almost zero at a temperature of 121 and 131 °C for 15 min. After 5 min at 131 °C, the Log CFU/g was about 3 in the case of *A. baumannii*, where it was almost zero for *E. coli* and *P. aeroginosa.*

Gram positive bacteria were found to be more resistant than Gram negative bacteria. It was found that Log CFU/g was found almost zero for *Staphylococcus aureus* and *Streptococcus pyogenes* after a contact time of 15 min and 30 min for 131 °C and 121 °C, respectively (Figure 4 and Figure 5). However, the Log CFU/g reduction was almost zero for *Bacillus subtilis* (*B. subtilis*) at 121 °C for 60 min and at 131 °C for 30 min (Figure 6). Further, the elevated temperature of 111 °C was not sufficient to obtain the optimum degree of Log CFU/g reduction in Gram positive bacteria, as we can see in Figure 4, Figure 5, Figure 6. 

The differences in the degree of inactivation between Gram negative and Gram positive bacteria were might be due to the different cell wall structures of the bacteria [29,30,31]. Studies have reported that the peptidoglycan layers of Gram-positive bacteria cell walls are much thicker than those of Gram-negative bacteria. Therefore, the Gram-positive cells are stronger and less likely to be broken both mechanically and chemically [30]. However, the optimum degree of inactivation was found for the Gram negative bacteria at 121 °C for 15 min, it was found for the Gram positive bacteria at 121 °C and 131 °C for 60 min and 30 min, respectively. Since clinical solid waste contains a great variety of Gram positive and Gram negative bacteria, therefore, the time-temperature profile of 121 °C and 131 °C for 60 and 30 min, respectively, was used for the subsequent studies. 

It is reported that steam sterilization can effectively sterilize the clinical solid waste with the inactivation of pathogenic microorganisms in the waste [28,32]. According to STAATT, acceptable technology for treating infectious clinical solid waste must reach inactivation level III, which is a Log10 reduction for the vegetative cells of 6 [23]. The preliminary experiments of the present investigation on sterilization of clinical solid waste also support this claim. Results show that steam sterilization by autoclave is capable of achieving about 8 Log reductions at a time-temperature profile of 121 °C for 60 min, as shown in Table 1. Hence, steam autoclaving can be used to sterilize CSW. Consequently, clinical waste recycle-reuse programs could be conducted, which minimize the waste load, and decrease labor and treatment costs. For this reason steam autoclaving of CSW has been considered as one of the alternatives to incineration technology in clinical waste management [23]. However, there has been no consideration of the unexpected factor of bacterial re-growth in the sterilized waste. 

The re-growth of bacteria was observed in sterilized samples, as shown in Table 3. A temperature-time profile of 121 °C for 60 min was used to sterilize the bacteria contaminated waste. The detection of re-growing bacteria was carried out through biochemical reaction and the respective API kits for the bacteria, as shown in Table 4 and Table 5. It was found that the Gram positive bacteria re-grow more than Gram negative bacteria. Among the Gram positive bacteria, the re-growth of *B. subtilis* was observed on the second day of sterilization, whereas for *Staphylococcus aureus* and *Streptococcus pyogenes* it was observed on day 3 of sterilization and the re-growth of *A. baumannii* and *E. coli* started at day 4, and for *P. aeroginosa* on day 6 of sterilization. The re-growth of bacteria found might be due to failure of the destruction of bacterial cell wall with complete protein coagulation during sterilization. Therefore, some of the bacteria are just injured during sterilization, and in the presence of sufficient nutrients they can grow again. 

**Table 3:** Re-growth of bacteria in sterilized waste.

Bacteria Time, day	0	1	2	3	4	5	6	7
*Escherichia coli*	×	×	×	×	×	√	√	√
*Psudomonas aeroginosa*	×	×	×	×	×	×	√	√
*Acinetobacter baumannii*	×	×	×	×	√	√	√	√
*Staphylococcus aurous*	×	×	×	√	√	√	√	√
*Streptococcus pyogenes*	×	×	×	×	√	√	√	√
*Bacillus subtilis*	×	×	√	√	√	√	√	√

×—Re-growth negative; √—re-growth positive.

**Table 4:** Detection of Gram negative bacteria from re-growing bacteria in sterilized waste.

Gram staining reaction	Oxidase Test	Fermentation Test	TSI Test	API Kit	Name of Bacteria
Gram negative-rod shaped	-ve	NLF	-ve	API 20NE	*Acinetobacter baumannii*
Gram negative-rod shaped	+ve	NLF	-ve	API 20NE	*Pseudomonas aeruginosa*
Gram negative-rod shaped	-ve	LF	+ve	API 20 E	*Escherichia coli*

-ve: Negetive; +ve: Positive; LF: Lactose fermentative; NLF: Non-Lactose fermentative.

**Table 5:** Detection of Gram positive bacteria re-growing bacteria in sterilized waste.

Gram staining reaction	catalase Test	API Kit	Name of Bacteria
Gram positive cocci	+ve	API 20 staph	*Staphylococcus aureous*
Gram positive cocci	-ve	API 20 strep	*Streptococcus pyogene*
Gram positive bacilli	+ve	API 50 CHB	*Bacillus subtilis*

The definition of the term ‘sterilization’ is the complete destruction of all living microorganism [33]. Although, steam sterilization can inactivate the microrganims, the fact that bacteria can re-grow defies the definition of sterilization. Therefore, autoclaving of CSW should not be considered as an alternative technology to incineration. Due to the re-growth of bacteria, recycle-reuse of waste materials is not possible. Further, the treated waste requires disposal using a secondary treatment option. Therefore, the present study strongly recommends considering the re-growth factor of microorganisms from the treated waste during the determination of CSW treatment technology. Besides, if an autoclave is used for treating infectious waste, the waste must disposed off within 2 days of treatment in order avoid possible infection by re-growing bacteria from the treated waste. 

## 4. Conclusions

This study was conducted with simulated clinical solid waste artificially contaminated with bacteria and treated by steam autoclave in order to determine possible alternatives to incineration technology in clinical solid waste management. It was found that the degree of inactivation of bacteria depended on contat time and temperature. However, a higher temperature required less contact time to obtain the optimum degree of bacterial inactivation. The optimum degree of inactivation for the Gram negative bacteria was found at 121 °C for 15 min, and for the Gram positive bacteria at 121 °C and 131 °C for 60 min and 30 min, respectively. Since clinical solid waste contains a great variety of bacteria, therefore the optimum experimental conditions to obtain an optimal degree of inactivation of bacteria were 121 °C and 131 °C for 60 and 30 min, respectively, which were used for further studies. The re-growth of bacteria was seen for all the studied bacteria 6 days after sterilization of the waste. Due to this unexpected re-growth of bacteria in the sterilized waste, we conclude that steam autoclave should not be considered as an alternative technology of incineration in clinical solid waste management. 
